# Monotocy and the evolution of plural breeding in mammals

**DOI:** 10.1093/beheco/araa039

**Published:** 2020-05-06

**Authors:** Dieter Lukas, Tim Clutton-Brock

**Affiliations:** 1 Department of Zoology, University of Cambridge, Cambridge, UK; 2 Department of Human Behavior, Ecology and Culture, Max Planck Institute for Evolutionary Anthropology, Leipzig, Germany

**Keywords:** life history, social competition, sociality, phylogenetic reconstruction

## Abstract

In many mammals, breeding females are intolerant of each other and seldom associate closely but, in some, they aggregate in groups that vary in size, stability, and kinship structure. Aggregation frequently increases competition for food, and interspecific differences in female sociality among mammals are commonly attributed to contrasts in ecological parameters, including variation in activity timing, the distribution of resources, as well as the risk of predation. However, there is increasing indication that differences in female sociality are also associated with phylogenetic relationships and with contrasts in life-history parameters. We show here that evolutionary transitions from systems where breeding females usually occupy separate ranges (“singular breeding”) to systems where breeding females usually aggregate (“plural breeding”) have occurred more frequently in monotocous lineages where females produce single young than in polytocous ones where they produce litters. A likely explanation of this association is that competition between breeding females for resources is reduced where they produce single young and is more intense where they produce litters. Our findings reinforce evidence that variation in life-history parameters plays an important role in shaping the evolution of social behavior.

## Teaser

How many offspring a mother has may determine how social she can be. Phylogenetic analysis shows that, among mammals, female sociality (aggregations of breeding females) has evolved more frequently in species where mothers produce single young than in species where they produce litters.

## Introduction

In many mammals, adult females compete for access to resources necessary to raise young and occupy separate ranges or territories during the breeding season—as in many rodents, insectivores, and nocturnal carnivores (Eisenberg 1983; Wolff and Sherman 2007; Clutton-Brock 2016). The ranges of breeding females in these “singular breeders” overlap to varying extents with those of neighboring females, as well as with those of males: in some, females defend exclusive territories against members of both sexes, whereas, in others, they share their territories with particular males but exclude other mature females; in some, there is extensive range overlap between neighboring females, whereas, in others, breeding females share their ranges with nonbreeding relatives and unrelated breeding males but forage separately; finally, in a few, breeding females associate with nonbreeding relatives of both sexes in cohesive groups as in the social mole-rats and some social mongooses (Jarvis 1981; Clutton-Brock 2016).

In other species, breeding females are more tolerant of each other and multiple breeding females share a common range and form groups that include multiple individuals that breed regularly (Lewis and Pusey 1997; Clutton-Brock 2016). Although a relatively small proportion of all mammals form groups of this kind, these systems predominate within some Orders, including ungulates, cetaceans, and primates (Clutton-Brock 2016; Ward and Webster 2016). In most “plural breeders,” groups usually consist of breeding females born in the same group, as well as a number of nonbreeding natal males that will eventually disperse to breed elsewhere, together with one or more immigrant males that father most of the young born in the group. In a small number of species, groups consist of immigrant breeding females together with one or more natal breeding males (Clutton-Brock 1989; Lukas and Clutton-Brock 2011). Intraspecific variation in group size is common (Richard 1974; Lott 1991) and, in some cases, groups in some populations usually contain multiple breeding females, whereas, in others, many contain a single breeding female (Schradin 2013). In many cases, this variation appears to reflect contrasts in population density, with larger numbers of breeding females in groups where population densities are higher.

Many comparative studies have explored the distribution of female group size in particular Orders of mammals and have shown that interspecific differences are related to variation in habitat use, feeding ecology, activity timing, and population density (Jarman 1974; Kaufmann 1974; Bradbury and Vehrencamp 1977; Clutton-Brock and Harvey 1977; Gittleman 1989; Wright 1999; Fisher and Owens 2000; Ebensperger 2001), as well as to contrasts in body size, longevity, litter size, and juvenile development (Eisenberg 1983; van Schaik and Kappeler 1993; Rubenstein and Abbot 2017; Smith et al. 2017). However, analyses of variation in female sociality seldom distinguish between groups that incorporate several breeding females (plural breeders) and groups that include a single breeding female (singular breeders). The distinction between total group size and the number of breeding females that aggregate is important for it is the extent to which breeding females aggregate that affects social and mating competition. When breeding females aggregate, the number of potential breeding partners that individual males can guard effectively is higher (Emlen and Oring 1977), potentially increasing the intensity of sexual selection on males (Kvarnemo and Ahnesjo 1996) and the extent to which breeding males aggregate with each other (Andelman 1986). The number of breeding females in a group also affects social interactions by influencing average levels of kinship between group members (Lukas et al. 2005; Lukas and Clutton-Brock 2018). Although contrasts in group size often reflect differences in the number of breeding females that associate with each other, the relationship between total group size and the number of breeding females per group is inconsistent (Rubenstein et al. 2016). For example, some of the largest stable social groups found in mammals occur in singular breeders, like naked mole-rats (*Heterocephalus glaber*) where colonies can include several hundred individuals but only a single female breed in each group (Jarvis 1981; Braude 2000).

Previous theories about the evolution of sociality among breeding females (plural breeding) have focused on its association with diurnal activity patterns and susceptibility to predation, as well as with reliance on resources where direct competition between individuals foraging in close proximity is not intense (Jarman 1974; Wrangham 1980). However, there are both theoretical and empirical reasons for expecting life-history parameters to also play an important role. Lactating females experience substantial increases in energetic requirements in all mammals and the energetic costs of raising young increase with litter size (Speakman 2008; Hamel et al. 2010; Hinde and Milligan 2011). As a result, competition between coresident breeding females for resources necessary to raise offspring is likely to be more intense in polytocous species (where females produce multiple young at once) than in monotocous ones (females produce single young). Comparative studies support this: for example, female infanticide appears to be more frequent in polytocous than monotocous species (Lukas and Huchard 2019). In addition, polytocy is usually associated with the production of relatively altricial infants that need to be maintained in a nest or burrow (Eisenberg 1983; van Schaik and Kappeler 1993). As a result, many polytocous mammals are central place foragers (Stephens and Krebs 1986), which increases the energetic costs of aggregation in breeding females and so may constrain the evolution of sociality in breeding females (Kappeler 1998). Phylogenetic comparisons also suggest that monotocy may facilitate the evolution of sociality. Although singular breeding and polytocy appear to have been the ancestral condition in many phylogenetic groups of mammals (Leutenegger 1979; Lukas and Clutton-Brock 2013; Werneburg et al. 2016), many of the mammalian taxa where plural breeding is common are monotocous—including the primates, ungulates, and cetaceans (Eisenberg 1983; Clutton-Brock 2016). Transitions to monotocy from polytocy appear to be rare and to have occurred at a relatively early stage in many mammalian lineages (Leutenegger 1979; Bordes et al. 2011; Werneburg et al. 2016; Battistella et al. 2019), whereas transitions in sociality appear to be more recent (Blomberg et al. 2003; Kamilar and Cooper 2013), suggesting that transitions to monotocy in these mammalian lineages might have removed constraints on the evolution of plural breeding.

Here, we use comparative data for mammals and phylogenetic reconstructions to investigate whether there is a consistent relationship between the evolution of monotocy and the distribution of plural breeding. Given that plural breeding is rarer and apparently the derived system, we investigated whether contemporary mammals in which plural breeding has been observed at all are more likely to be monotocous, whereas species in which only singular breeding occurs are more likely to be polytocous. To test the prediction that transitions to the production of single offspring remove constraints on aggregation in breeding females, we subsequently investigated whether any transitions to plural breeding have been more common in monotocous lineages than in polytocous ones.

## Material and Methods

### Data and classifications

We based the classification of the social system of mammals on our previous databases (Lukas and Clutton-Brock 2013, 2017a) and excluded the few mammalian species in which females produce eggs. We identified species as monotocous if median litter size was less than 1.5 and as polytocous if it was greater than 1.5. Data for litter size (number of young per birth, central tendency across females in wild populations), for adult body mass (female mass in grams, central tendency across females in both captive and wild populations), for diurnality (whether a species is strictly nocturnal or whether part of its activity occurs during the day), and for diet (whether a species is a herbivore, carnivore, or omnivore) were obtained from a combination of primary and secondary sources, including published databases (Carey and Judge 2002; Ernest 2003; Bielby et al. 2007; McCarthy et al. 2008; de Magalhaes and Costa 2009; Jones et al. 2009; Wilman et al. 2014). If entries in these databases differed slightly, we used the median value and, if entries were substantially different, we referred to the primary literature to identify the most likely value.

We restricted species in our analysis to those for which field-based reports of social behavior were available (following Schradin 2017; Lukas and Clutton-Brock 2017b). We identified species as singular breeders if most females occupy separate home ranges or territories during the breeding season (even if they share these with nonbreeding females or with males) and as plural breeders if multiple breeding females share a common range and actively associate with each other (Ward and Webster 2016), forming groups that usually include more than one individual that breeds regularly. Our definition of plural breeding includes fission–fusion species, such as chimpanzees where females may spend part of the day foraging independently but live in discrete communities that include multiple breeding females that share a common range. We classified species that live in mixed sex pairs or in groups where only a single female breeds regularly (like the social mole-rats, callitrichid primates, and several social mongooses) as singular breeders. We focus our classification of sociality on the behavior of breeding females, those that are in the later stages of pregnancy or have dependent young and do not consider the behavior of juveniles, males, or adult females who are not breeding. In some species where breeding females commonly aggregate, some breeding groups include a single breeding female, whereas others include several females that breed regularly (Nievergelt et al. 2002; Dalerum 2007; Schradin et al. 2012; Weidt et al. 2014; Valomy et al. 2015; Agnani et al. 2018; Miles et al. 2019). Where intrapopulation variation of this kind was reported, we classified species as plural breeders if, throughout the breeding season, most breeding females are found in groups where several females breed regularly and as singular breeders if the majority of breeding females were found in groups that included a single breeding female. We used a majority rule to reduce risks of misclassification of rare observations that are likely to be nonadaptive (following Schradin et al. 2018). In the small number of species where studies of different populations have shown that plural breeding predominates in some populations or at some times, whereas singular breeding predominates in others, often in association with relatively low population density (e.g., striped mice; Schradin et al. 2012), we classify them as plural breeders if plural breeding predominates in either population as this is the rarer and derived system. It has been suggested that, where multiple records of female group size are available, they should be included as separate points (e.g. Miles et al. 2019). However, like most other comparative studies, we preferred to include a single value for each species in order to avoid particular species weighting our analyses disproportionately. We also generally do not have matching data on litter size for each population, so we cannot attempt to explain the full variation in sociality across populations; we do not know the history of the populations within a given species, so we cannot infer if and how often transitions in sociality might have occurred (Stone et al. 2011). The number of species for which multiple records showing differences in female sociality are available is relatively low and whether they are classified as plural or singular breeding does not appear to affect the outcome of our analyses.

For our phylogenetic reconstructions, we relied on a mammalian supertree (Rolland et al. 2014) and did not resolve polytomies or modify the branch lengths in any analyses. All data and sources are deposited at the Knowledge Network for Biocomplexity (doi: 10.5063/F1P8497S).

### Statistical approaches

To test whether singular and plural breeders differ in their life-history parameters, we ran binomial regression models using MCMCglmm (Hadfield and Nakagawa 2010) in the statistical software R (R Core Team 2019). We first investigated whether differences in monotocy/polytocy are associated with the distribution of plural breeding. We included the phylogenetic relationship between species as covariance matrix, set a flat prior (Hadfield 2010) and used 1 500 000 iterations, a burn-in of 500 000 and a thinning interval of 10. Each analysis was repeated three times and visually inspected for convergence. We report the 95% confidence intervals (CIs) based on the Bayesian sample for all relationships to determine whether an estimated effect is systematically different from 0. The proportion of the Bayesian sample that crosses 0 is similar to a *P*-value estimate; in all our cases, the CI did not contain 0 suggesting that the results are robust.

For the phylogenetic reconstruction, we first estimated the strength of the phylogenetic signal for polytocy/monotocy and singular/plural breeding using the function phylosig in PhyTools (Revell 2012) in R to calculate the *K*-statistic and lambda, assessing their significance by comparison to 10 000 simulations. To assess whether monotocy and plural breeding coevolved, we performed reconstructions using the function Discrete in BayesTraits V3 (Pagel et al. 2004). We ran models assuming either an independent or a dependent evolution, estimating the parameters using maximum likelihoods based on 100 tries per model specification.

## Results

**Figure 1:**
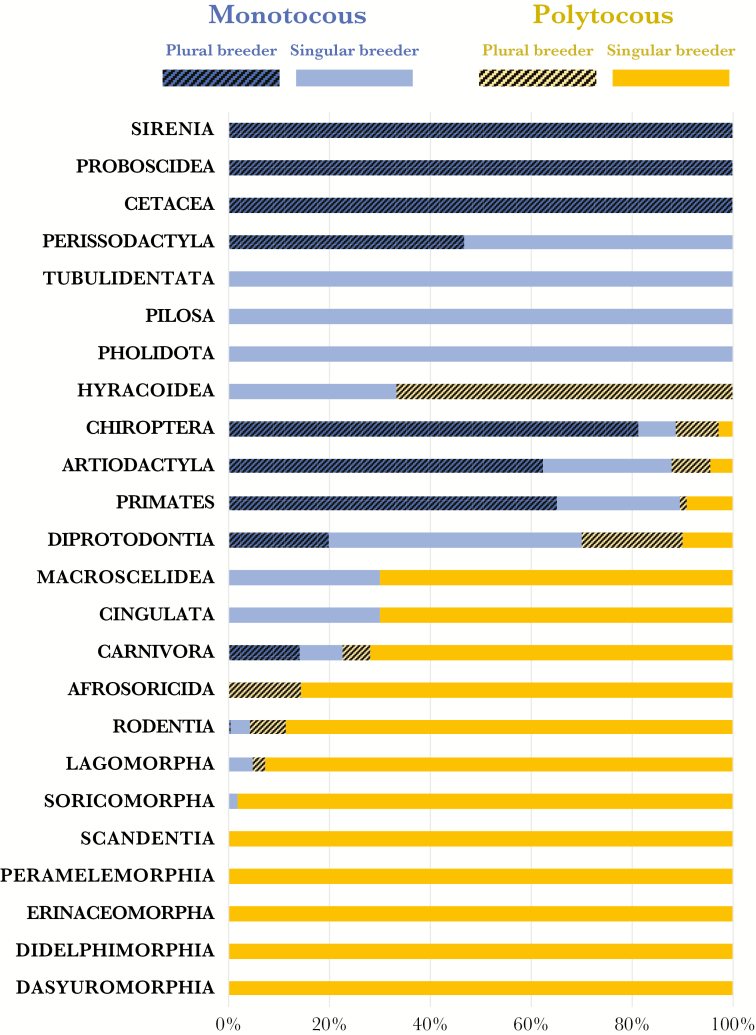
Proportion of species in mammalian Orders that records show to be either monotocous (in blue) or polytocous (in yellow) and either plural (dark shading) or singular breeders (plain colour). In Orders in which the majority of species are monotocous, such as cetaceans or primates, plural breeding is more likely than in Orders in which most species are polytocous, such as carnivores or rodents.

In the 1267 species in our sample, 44% were classified as monotocous and 56% as polytocous. In 54% of the mammalian Orders represented in our sample, all species were either monotocous or polytocous, whereas 46% of Orders included both monotocous and polytocous species. Polytocy is predominant (more than 75%) in 10 of the 24 mammalian Orders represented in our sample, whereas monotocy predominates in another 10 (see Figure 1).

Although 56% of contemporary mammals in our sample are polytocous, more than 85% of all species classified as plural breeders in our sample are monotocous and, in all seven Orders of mammals where plural breeding is common (artiodactyls, cetaceans, bats, diprotodons, perissodactyls, proboscidea, and primates), most or all species are monotocous (see Figure 1). Compared with species that we classified as singular breeders (and controlling for phylogenetic effects), plural breeding mammals have significantly smaller litter sizes (mean effect of larger litter size: −118.0, 95% CI: −168.7 to −76.5; 810 singular vs. 457 plural breeders): among singular breeders, median litter size is 3 and more than 80% of species produce two or more offspring per breeding attempt. In contrast, among species classified as plural breeders, median litter size is one and only 14% of species produce two or more offspring per breeding attempt.

The influence of litter size on the distribution of plural breeding is independent of whether a species is nocturnal or diurnal (effect of larger litter size on occurrence of plural breeding: −25.4, 95% CI: −33.1 to −16.5; effect of being nocturnal rather than diurnal: −8.9, 95% CI: −13.2 to −4.7; 374 singular vs. 115 plural breeders) and whether it is omnivorous, herbivorous, or carnivorous (effect of larger litter size on occurrence of plural breeding: −19.7, 95% CI: −27.8 to −11.4; effect of being herbivore rather than carnivore or omnivore: 7.7, 95% CI: 0.5 to 15.7; 409 singular vs. 333 plural breeders). In a combined model, litter size also has a stronger estimated effect on the distribution of plural breeding than body mass when both factors were standardized by subtracting each species’ value from the mean across species divided by the standard deviation (effect of larger litter size on occurrence of plural breeding: −11.4, 95% CI: −16.0 to −7.6, effect of larger body size: 3.3, 95% CI: 1.4 to 5.4; 793 singular vs. 457 plural breeders).

Phylogenetic reconstructions provide further evidence of an association between the evolution of plural breeding and monotocy. Our phylogenetic reconstructions support previous evidence that the ancestor of mammals was polytocous and singular breeding (Lukas and Clutton-Brock 2013; Werneburg et al. 2016). They show that the prevalence of monotocy or polytocy is comparatively stable within phylogenetic groups, with few evolutionary transitions between monotocy and polytocy necessary to explain the distribution of monotocy among the species in our sample (phylogenetic signal: *K* = 0.74; lambda 0.98; both *P* < 0.001). In contrast, plural breeding is more labile and, in several phyla, appears to have originated relatively recently (phylogenetic signal: *K* = 0.45; lambda 0.95; both *P* < 0.001).

The evolution of plural breeding has occurred between 10 and 20 times more frequently in monotocous than in polytocous lineages (see Supplementary Material) and models assuming that monotocy and plural breeding evolved independently received consistently less support than those indicating that the two traits are associated (median of log-likelihoods of independent models: −1105; median of log-likelihood of dependent models: −829). All models that we explored suggest that evolutionary transitions from polytocy to monotocy occur before transitions from singular to plural breeding and that transitions to monotocy increase the probability of subsequent transitions from singular to plural breeding. The most likely dependent model suggests that monotocy evolved in singular breeders and was only lost in relatively few instances in plural breeders. Restricting the phylogenetic reconstructions to assume that the evolution of plural breeding was equally likely in monotocous and polytocous lineages did not change the inference that all monotocous plural breeders originated from monotocous singular breeders: the model suggested that there have been no transitions to monotocy in plural breeders, with the only change in the model inference being a 10-fold increase in the rate at which plural breeding would be lost in polytocous species. A model which restricts both gains and losses of plural breeding to occur with equal probability in polytocous and monotocus species infers the ancestral state to be monotocous and has a much lower likelihood than the model assuming that transitions to plural breeding are more likely to occur in monotocous species.

## Discussion

Our analysis supports the suggestion that interspecific contrasts in sociality among breeding females are associated with variation in litter size and with the evolution of monotocy. Although 65% of all mammals are polytocous and polytocy is likely to have been the original ancestral condition for all live-bearing mammals (Myhrvold et al. 2015; Werneburg et al. 2016), more than 85% of species where breeding units usually include more than one female that breeds regularly are monotocous. Plural breeders produce smaller litters than singular breeders and transitions from singular breeding to plural breeding appear to have been substantially more frequent in monotocous lineages than in polytocous ones. Although plural breeding and monotocy are both associated with diurnal activity, this association does not account for the association between plural breeding and monotocy, which persists when the effects of differences in activity timing are controlled.

A likely reason for the association between plural breeding and monotocy is that energy requirements of breeding females (especially during lactation) are substantially higher in polytocous species, generating more intense competition between females for the resources necessary to provision and raise offspring (Johnson et al. 2001; Speakman 2008). Although female interference in breeding attempts by other females is not confined to polytocous species, it appears to be more common in polytocous than monotocous species: for example, physiological suppression of fertility in subordinate and female infanticide have been recorded to occur more commonly in polytocous species than in monotocous ones (Clutton-Brock 2016; Lukas and Huchard 2019).

The association between monotocy and plural breeding may also help to explain contrasts in social behavior between major taxa—and the nature of social relationships in particular. Comparative studies show that rates of competitive interactions among group members tend to be reduced in mammals where average levels of kinship between group members are relatively high and more common when average kinship is low. In addition, costly forms of asymmetrical or altruistic cooperation, such as provisioning young born to others, are largely confined to species with high levels of kinship and rare where average kinship between group members is low (Lukas and Clutton-Brock 2018).

The association between plural breeding and monotocy raises questions about the evolution of monotocy itself. Within mammals, the evolution of monotocy often appears to be associated with the need for precociality in infants—either because they need to cling to their mothers or to the substrate (as in primates and bats) because they need to be able to locomote independently within a few hours of birth (as in many ungulates and cetaceans) or because young are exposed to potentially high levels of predation and there is a need to minimize the duration of the period of early development (as in the pinnipeds) (Martin and MacLarnon 1985; Kappeler 1998; Hamilton et al. 2011). However, it is likely that the ecological circumstances favoring monotocy differ between major animal groups (Promislow and Harvey 1990; Charnov 1991; Tökölyi et al. 2014) and this is too large a topic to consider in detail here.

Our analysis illustrates the way in which contrasts in life-history parameters can influence the evolution of sociality and the form of social behavior. Monotocy and the production of precocial young may allow members of some species to occupy niches or habitats where altricial young could not be reared but may, at the same time, preclude the evolution of some breeding systems. For example, among mammals, cooperative breeding systems, where group members other than their parents are principally responsible for guarding and feeding infants, are restricted to polytocous species, where average kinship between group members is relatively high (Lukas and Clutton-Brock 2012). This may be because, in monotocous species, helpers cannot generate large effects on the reproductive output of breeders or because monotocy reduces average kinship between group members to low levels, in particular in large groups, precluding the evolution of breeding systems involving costly forms of cooperation. The role of litter size in the evolution of breeding systems may also help to explain differences in the distribution of breeding systems outside of mammals. In birds and insects, where females usually produce multiple eggs (de Maghalaes and Costa 2009), cooperative and eusocial breeding appears more common than plural breeding (Riehl 2013; Koenig and Dickinson 2016; Rubenstein and Abbot 2017).

The effects of phylogenetic contrasts in life-history patterns emphasize the need for comparative studies to focus at the most appropriate taxonomic level. Where related species differ in their life-history parameters, analyses of the distributions of traits within Orders can offer important insights into relationships between contrasts in social behavior and variation in ecology and life-history parameters (Rubenstein 1989; Kappeler and Pereira 2003). However, where all members of the same Order share similar life-history characteristics, comparisons may need to span different radiations in order to identify the extent to which life-history parameters facilitate or constrain the evolution of social behavior (Harvey and Pagel 1991; Rubenstein and Abbott 2017). For example, although there have been multiple analyses of the distribution of sociality in higher primates (Clutton-Brock and Harvey 1980; Wrangham 1980; Sterck et al. 1997; Shultz et al. 2011), the importance of monotocy on the evolution of sociality has (apparently) not been previously recognized because a high proportion of species are both monotocous and plural breeding.

Finally, our analysis shows how differences in ecology, life history, and phylogeny are likely to interact in their effects on breeding systems and social organization. Although it is sometimes suggested that phylogenetic relationships, rather than contrasts in ecology, control contrasts in social behavior between higher level taxa (Gittleman 1986; Shultz and Dunbar 2007), contrasts in life-history parameters between major taxonomic groups are likely to represent ecological adaptations and to be maintained by selection (Williams 1966; Lack 1968; Stearns 2000). Relationships between ecological variation and contrasts in social organization and reproductive strategies may consequently be less direct and may vary more widely between taxa than was recognized in early comparative studies. However, this variation does not contradict the view that contrasts in ecology play a central role in guiding the evolution of interspecific differences in social behavior and breeding systems.

## Supplementary Material

araa039_suppl_Supplementary_Material

araa039_suppl_Supplementary_Data
