# Supplementary material for: Monotocy and the evolution of plural breeding in mammals
Source: Behav Ecol. 2020 May 6;31(4):943–9. doi: 10.1093/beheco/araa039 (PMC7390990; doi:10.1093/beheco/araa039)
Supplement: araa039_suppl_Supplementary_Material [file araa039_suppl_supplementary_material.docx]

**Supplementary Figure 1: Transition probabilities in the model assuming a dependent evolution between breeding system and litter-size**

The figure depicts the four trait combinations between breeding system (singular versus plural breeding) and litter-size (polytocy versus monotocy), and the likelihood of transitions between them. The most likely ancestral state for the mammalian species in our sample is singular breeding and polytocy. Only few transitions towards females being monotocous are likely to have occurred, but since they occurred early in some lineages, many extant species are monotocous (601 out of 1267 species in our sample are monotocous). Transitions to plural breeding are around 10-20 times more likely to have occurred in monotocous than in polytocous lineages. Plural breeding appears unstable, and is frequently lost.
